# Classification of Takifugu rubripes, *T*. *chinensis* and *T*. *pseudommus* by genotyping-by-sequencing

**DOI:** 10.1371/journal.pone.0236483

**Published:** 2020-08-27

**Authors:** Yeon Jung Park, Mi Nan Lee, Jae Koo Noh, Eun Soo Noh, Jung Ha Kang, Jung Youn Park, Eun Mi Kim

**Affiliations:** Biotechnology Research Division, National Institute of Fisheries Science, Busan, Korea; University of Cincinnati, UNITED STATES

## Abstract

*Takifugu rubripes* is more expensive than other species of the genus because of its high protein content and special flavor. However, it is easily confused with imported *T*. *chinensis* and *T*. *pseudommus* because they have similar morphological characteristics. We identified single nucleotide polymorphism (SNP) markers of *T*. *rubripes* by genotyping-by-sequencing (GBS) and evaluated their ability to distinguish among *T*. *rubripes*, *T*. *chinensis*, and *T*. *pseudommus*. In all, 18 polymorphic SNPs were subjected to phylogenetic analyses of the three *Takifugu* species. Additionally, we subjected a second set of samples to Sanger sequencing to verify that the polymorphic SNPs could be used to evaluate the genetic variation among the three *Takifugu* species. A phylogenetic tree that included the analyzed sequence of set A, which is referred to as the reference sequence, and a validation sequence of set B with 18 SNPs were produced. Based on this phylogenetic tree and STRUCTURE analyses, *T*. *rubripes*, *T*. *chinensis* and *T*. *pseudommus* have low genetic variation and should be considered the same gene pool. Our findings suggest that further studies are needed to estimate the genetic association of the three *Takifugu* species.

## Introduction

The family Tetraodontidae comprises 187 species of 28 genera, among which 33 species of 10 genera have been reported in the Republic of Korea (ROK) [1, 2]. Puffer fish of the genus *Takifugu* (Tetraodonitiformes, Tetraodontidae) are distributed mainly in the Western Pacific Ocean. These are valuable fish species and about 2,200–3,800 tons are caught wild and over 8,000 tons are imported for consumption in the ROK annually [3]. Although several *Takifugu* species are consumed in East Asia, *T*. *rubripes* is the most economically important due to its special flavor [4]. However, the domestic wild catch of *T*. *rubripes* has dramatically decreased due to overfishing, spawning ground destruction, and contamination. To meet demand, this species is imported from China, India, and Japan live or in a frozen, dry form (http://www.mof.go.kr). The identification and classification of these fish species had raised some controversy. Especially, *T*. *rubripes*, *T*. *chinensis*, and *T*. *pseudommus* have the same habitat and similar morphological characteristics [5]. Comparison of their meristic characters indicates overlapping morphological characteristics, such as the number of dorsal, vertebral, anal, and pectoral fin rays [6]. *T*. *rubripes*, unlike *T*. *chinensis* and *T*. *pseudommus*, has irregular round black spots and white patterns in front of the caudal fin on the sides of the body. *T*. *chinensis* has no such black marks while *T*. *pseudommus* has white spots scattered on a black background on the dorsal and lateral sides of the body [2, 7]. However, species identification based on external evidence can lead to controversy. In addition, species identification is hampered by distribution in the form of processed products, such as pellets, sashimi, cooked food, and puffer-fish skin. Therefore, the relationships among *T*. *rubripes*, *T*. *chinensis* and *T*. *pseudommus* must be evaluated using other characteristics.

DNA-based identification methods, including molecular markers, have been applied to identify the various marine species [8]. The used technique for identification was performed in the pacific tuna using allozyme [9], the horse mackerel using mitochondrial DNA (mtDNA) [10], the Indian sciaenidsr using randomly amplified polymorphic DNA (RAPD) [11], the gadoid using restriction fragment length polymorphism (RFLP) [12]. Of the nuclear single locus, microsatellites or simple sequence repeats (SSRs) have been used for individual and population genetics because of their high abundance, variability, and neutrality [13]. For instance, polymorphic loci have been used to distinguish *T*. *rubripes* from *T*. *chinensis* and *T*. *pseudommus* [14]. Although microsatellites facilitate examination of genetic variation and genetic structure, tools that use such markers have disadvantages such as inconsistencies in allele size calling caused by use of automatic sequencers and errors in size determination [15]. Among the various types of markers in use, single nucleotide polymorphisms (SNPs) are the most abundant in a genome and the most suitable for identification and genotyping [16]. Genotyping-by-sequencing (GBS) enables the identification of SNPs for high-throughput screening [17]. Compared to next-generation sequencing (NGS), GBS is a simpler and more rapid method for generating SNP data [18]. It has important advantages such as simplified library preparation, high-level genome coverage, and cost effectiveness if an appropriate restriction enzyme is used [17]. It has been used for discovery genome mapping, quantitative trait loci (QTL) analyses, and genetic stock identification for marine species such as the Chinook salmon *Oncorhyncus tshawytscha* and the three-spine stickleback *Gasterosteus aculeatus* [19, 20]. No study of the genetic diversity of marine organisms based on SNPs and GBS has been reported.

In this study, we developed a new set of SNPs by GBS to evaluate the genetic variation among *T*. *rubripes*, *T*. *chinensis*, and *T*. *pseudommus*. To enhance our understanding of the genetic relationships among these three species, we created a phylogenetic tree and analyzed the population structure. We also estimated the reproducibility of the GBS results for species identification. These results will facilitate the identification of these three morphologically similar species.

## Material and method

### Sample collection and DNA extraction

Refrigerated fish were purchased at the cooperative fish market from May 2018. These samples were sent to the Marine Fish Resource Bank of Korea at Pukyong National University for morphological identification by a specialist and deposit as reference samples. The 96 samples of set A included 38 of *T*. *rubripes*, 28 of *T*. *chinensis*, and 30 of *T*. *pseudommus* and were used to identify SNPs by GBS. The samples included wild *T*. *rubripes* from Sinan-gun, Incheon in Republic of Korea (ROK), cultured *T*. *rubripes* from China and Japan; wild *T*. *chinensis* from Mokpo, Gunsan in ROK; and wild *T*. *pseudommus* from Boryeong, Mokpo in ROK (Table 1). Sample set B, which included wild *T*. *rubripes* from Sinan and Incheon, was used to verify the SNPs.

**Table 1:** Collection of the *Takifugu* samples used in genetic analyses.

Species	Locality	Sampling Date	Sample size	Status	
*Takifugu rubripes*	Japan (Shimonoseki)	2018.05.09	30	Culture	Set A
	China (Shandong, Weihai)	2018.05.09	3	Culture	
	Korea (Sinan, Imjado)	2018.05.17	3	Wild	
	Korea (Incheon)	2018.05.15	2	Wild	
*Takifugu chinensis*	Korea (Mokpo, Gunsan)	2018.05.15	28	Wild	
*Takifugu pseudommus*	Korea (Mokpo, Boryeong)	2018.05.15	30	Wild	
*Takifugu rubripes*	Korea (Sinan, Imjado)	2018.05.17	22	Wild	Set B
	Korea (Incheon)	2018.05.15	23	Wild	

Pieces of fin from raw fish samples were fixed in 99% alcohol and genomic DNA was extracted using the DNeasy 96 Blood & Tissue Kit (Qiagen) according to the manufacturer’s instructions. DNA was quantified using a NanoDrop ND-1000 instrument (Thermo Fisher, Barrington, IL). The DNA concentration was normalized to 10 ng/μL and used for library preparation.

### Analysis of COI gene

To analyze genetic variation among the three *Takifugu* species, the polymerase chain reaction (PCR) products of the cytochrome oxidase subunit I (COI) gene in mitochondrial DNA were obtained using the universal primers VF2 (5′-TCA ACC AAC CAC AAA GAC ATT GGC AC-3′) and FishR2 (5′-ACT TCA GGG TGA CCG AAG AAT CAG AA-3′). The PCR mixture consisted of 1× PCR buffer, 250 μM each dNTP, 2.5 U Ex-Taq DNA polymerase (TaKaRa), 20 ng DNA sample, and 10 pmol universal primers in a total volume of 20 μL. PCR amplification was performed as follows: 10 min of denaturation at 95°C, 35 cycles (45 s at 95°C, 45 s at 56°C, 1 min at 72°C) of amplification, and a final extension for 5 min at 72°C using an ABI 2729 Thermocycler. PCR products were visualized by gel electrophoresis and purified using an Expin PCR SV (GeneAll, ROK). COI sequences were amplified by PCR using the Big Dye Terminator v. 3.1 Cycle Sequencing Kit (Applied Biosystems) and ABI 3730XL DNA analyzer (Applied Biosystems, Waltham, MA).

### High-quality library production

The *in silico* digestion was performed with *Ape*KI, *Pst*I, and *Ape*KI-*Pst*I to select the appropriate enzyme for the *T*. *rubripes* DNA. GBS libraries were constructed using *Ape*KI (GCWGC) and based on a protocol modified from Elshire et al. [17]. The oligonucleotides consisting of a common adapter and the upper and lower strands of each barcode adapter were diluted individually in 50 μM TE and annealed in a thermocycler (95°C for 2 min; ramp down to 25°C at 0.1°C/s; 25°C for 30 min; 4°C hold). Barcodes and common adapters were diluted in 10× adapter buffer (500 mM NaCl, 100 mM Tris-Cl) to 10 μM, mixed at a one-to-one ratio, and then 2.4 μL was added to a 96-well PCR plate. DNA samples (100 ng/μL) were added to the wells containing the individual adapters. The samples (DNA plus adapters) were digested at 75°C with *Ape*KI (New England Biolabs, Ipswich, MA) overnight. Adapters were ligated to sticky ends by adding 30 μL solution containing 10× ligase buffer and T4 DNA ligase (200 units) to each well. The samples were incubated at 22°C for 2 h and 65°C for 20 min to inactivate T4 DNA ligase. Sets of 96 digested DNA samples, each using a different barcode adapter, were combined (5 μL each) and purified using a QIAquick PCR Purification Kit (Qiagen) following the manufacturer’s protocols and eluted in a final volume of 50 μL. The amplified restriction fragments from each library were added to 50 μL of a mixture of 2 μL pooled DNA fragments, Herculase II Fusion DNA Polymerase (Agilent), and 25 pmol of the following primers: (A) 5′-AAT GAT ACG GCG ACC ACC GAG ATC TAC ACT CTT TCC CTA CAC GAC GCT CTT CCG ATC T-3′ (58 mer) and (B) 5′-CAA GCA GAA GAC GGC ATA CGA GAT CGG TCT CGG CAT TCC TGC TGA ACC GCT CTT CCG ATC T-3′ (61 mer). PCR was performed using a Life ECO Thermal Cycler (Bioer Technology Co.) with the following parameters: initial denaturation at 95°C for 2 min, 16 cycles (95°C for 30 s, 62°C for 30 s, and 68°C for 30 s) of amplification, and a final extension at 68°C for 5 min. The amplified sample pools represented a sequencing library. The libraries were purified as above, except that the final elution volume was 30 μL. To evaluate the amplified fragment size, 2 μL was loaded onto an 1.5% agarose gel with running conditions of 200V for 25min and library quality was checked using an Agilent Tape station with a high-sensitivity DNA chip.

### Preprocessing

Raw sequences were de-multiplexed using the barcode sequences and trimmed using an *SEEDERS in-house* python script [21]. While filtering reads that contain ambiguous bases in the barcode, this script divides the raw Illumina FASTQ file into the 96 individual FASTQ files based on the barcode sequences. Reads were also trimmed using the cutadapt version 1.8.3 [22] if the sequence contained the common adapter.

The de-multiplexed reads were trimmed using DynamicTrim and LengthSort programs in the Solexa QA package v.1.13 [23]. The sequence near the primer that had Phred quality score fell below Q = 20 (or error probability 0.05) were removed. In addition, 5′ and 3′ stretches of ambiguous ‘N’ nucleotides were trimmed. Finally, poor-quality sequence reads, along with the DynamicTrim phred score was <20 bases and the LengthSort process used a short read length of <25 bases [23], were discarded.

### Alignment & detection of SNP and InDel

Burrows-Wheeler Aligner (BWA, 0.6.1-r104) software was used to align the clean reads to the reference genome [23]. The BWA default values for mapping were used, except for the following: seed length (-l) = 30, maximum differences in the seed (-k) = 1, number of threads (-t) = 16, mismatch penalty (-M) = 6, gap open penalty (-O) = 15, gap extension penalty (-E) = 8. Mapped reads were extracted from the resulting BAM file using SAMtools v. 0.1.16 for further analyses [24]. The high mapping quality assured reliable mapping of the reads, which is important for variant calling. SNPs were called only at the variable positions with a minimal mapping quality (Q) of 30 using the varFilter command. The minimum and maximum read depths were 3 and 95, respectively. A *SEEDERS in-house* python script considering biallelic loci was used to select significant SNPs from the called SNPs positions [25]. Depending on the ratio of SNP/InDel reads to mapped reads, the variant type was classified into three categories: homozygous SNP/InDel for > 90%, heterozygous SNP/InDel for 40–60%, and other [21, 26]. To control the quality of markers, a missing proportion (MSP) < 0.3 and a minor allele frequency (MAF) > 0.1 were selected [27].

### Experimental validation of SNPs

Common SNPs were selected by comparing the nucleotide sequences of the SNP loci of the samples of the same variety, and the polymorphic SNP loci were selected by comparing common SNPs among the varieties. When selecting common SNPs in the same variety, ≤50% missing data were allowed.

A subset of selected polymorphic SNPs was used for validation by Sanger sequencing. Primers were designed to produce 500–600 bp amplicons containing at least one putative SNP using Primer3 v. 2.3.5 [28]. Each 20 μL PCR reaction comprised 1× PCR buffer, 2.5 U Ex-Taq DNA polymerase (TaKaRa), 250 μM each dNTP, 10 pmol each SNP marker, and 20 ng genomic DNA template. PCR was performed using an ABI 2720 Thermocycler as follows: 10 min of denaturation at 95°C, 35 cycles (45 s at 94°C, 45 s at 58°C, and 45 s at 72°C) of amplification, and a final extension for 5 min at 72°C. The PCR products were assessed by 2% agarose gel electrophoresis and purified with Expin PCR SV (GeneAll Biotechnology, Seoul, ROK). The purified PCR products were amplified using an ABI BigDye Terminator v. 3.1 Cycle Sequencing Kit (Applied Biosystems), and run on an ABI 3730XL DNA analyzer (Applied Biosystems). The acquired bidirectional sequences and the reference sequence were aligned in SeqMan software (DNASTAR).

### Data analysis

The COI sequences were assembled using ClustalW multiple alignments with BioEdit software v. 7.2 (http://www.mbio.ncsu.edu/bioedit/bioedit), and haplotypes and polymorphisms were analyzed using DnaSP v. 5.1 [29]. Genetic relationships were inferred by the maximum-likelihood method with 1,000 replications based on the Tamura-Nei model using MEGA v. 6 [30]. Analyses of population genetic structure were assessed to SNP dataset by Bayesian model-based cluster analyses in STRUCTURE v. 2.3.4 [31]. The analyses were based on the admixture and correlated allele frequencies models. Clusters (K) were estimated in five replicates using K values of 1 to 10, 10,000 of burn-in period and 10,000 iterations for each run. STRUCTURE analyses were performed on the polymorphic SNP dataset of *T*. *rubripes*, *T*. *chinensis*, and *T*. *pseudommus* individuals to evaluate the population structure of these three species.

## Results

### Genetic variation of COI gene

The 141 individuals of the three *Takifugu* species were identified as two or more species based COI sequence of *T*. *rubripes* (Acc. No. AP006045), *T*. *chinensis* (KY514072), or *T*. *pseudommus* (KY514075). Sequence analyses of the 600 bp COI gene fragment identified 15 polymorphic sites with sequence variation, which resulted in 10 haplotypes (Table 2). There were seven, six, and seven haplotypes in the *T*. *rubripes*, *T*. *chinensis*, and *T*. *pseudommus* samples, respectively. Haplotype analyses demonstrated no variation in alleles among *T*. *rubripes*, *T*. *chinensis*, and *T*. *pseudommus*. Most individuals of the three *Takifugu* species were of Hap_1. The 30 individuals of the Shimonoseki cultured population were of one haplotype, which had a nucleotide diversity of 0%.

**Table 2:** Variable nucleotide sites among the three *Takifugu* speices used for SNP development and validation.

Polymorphic sites																											*T*. *rubripes*				*T*. *chinensis*	*T*. *pseudommus*
																											*Korea*		*China*	*Japan*	*Korea*	*Korea*
		16		62		93		105		149		167		270		299		305	312	386	392	398		455		473	Incheon	Sinan	Weihai	Shimonoseki	Gunsan	Boryeong
Hap_1		G		C		G		G		T		A		G		T		A	G	T	A	G		A		A	15	14	3	30	14	20
Hap_2		.		.		.		.		.		.		.		.		.	.	C	.	.		.		G	2	2			1	
Hap_3		.		.		.		.		.		G		A		.		.	.	.	G	A		.		.	1					
Hap_4		.		.		.		.		.		.		A		.		.	.	C	.	.		.		G	1				1	1
Hap_5		.		.		.		.		.		.		A		.		.	.	.	.	.		.		.	2				3	2
Hap_6		.		.		.		.		.		.		.		.		.	.	.	.	A		.		.	2	2			2	4
Hap_7		.		.		.		.		.		.		.		.		G	.	.	.	.		.		.		1				
Hap_8		.		.		.		.		.		.		.		.		.	.	.	.	.		G		.					1	1
Hap_9		.		.		.		.		.		.		.		A		.	.	.	.	.		.		.						1
Hap_10		T		G		C		C		G		.		A		.		.	C	.	.	.		.		.						1
																	Number of haplotypes										6	4	1	1	6	7
																	Haplotype diversity										0.573	0.456	0	0	0.589	0.547
																	Number of polymorphic sites										6	4	0	0	5	12
																	Nucleotide diversity(%)										0.198	0.117	0	0	0.154	0.191

### *In silico* genome digestion and sequencing raw data

**Fig 1:**
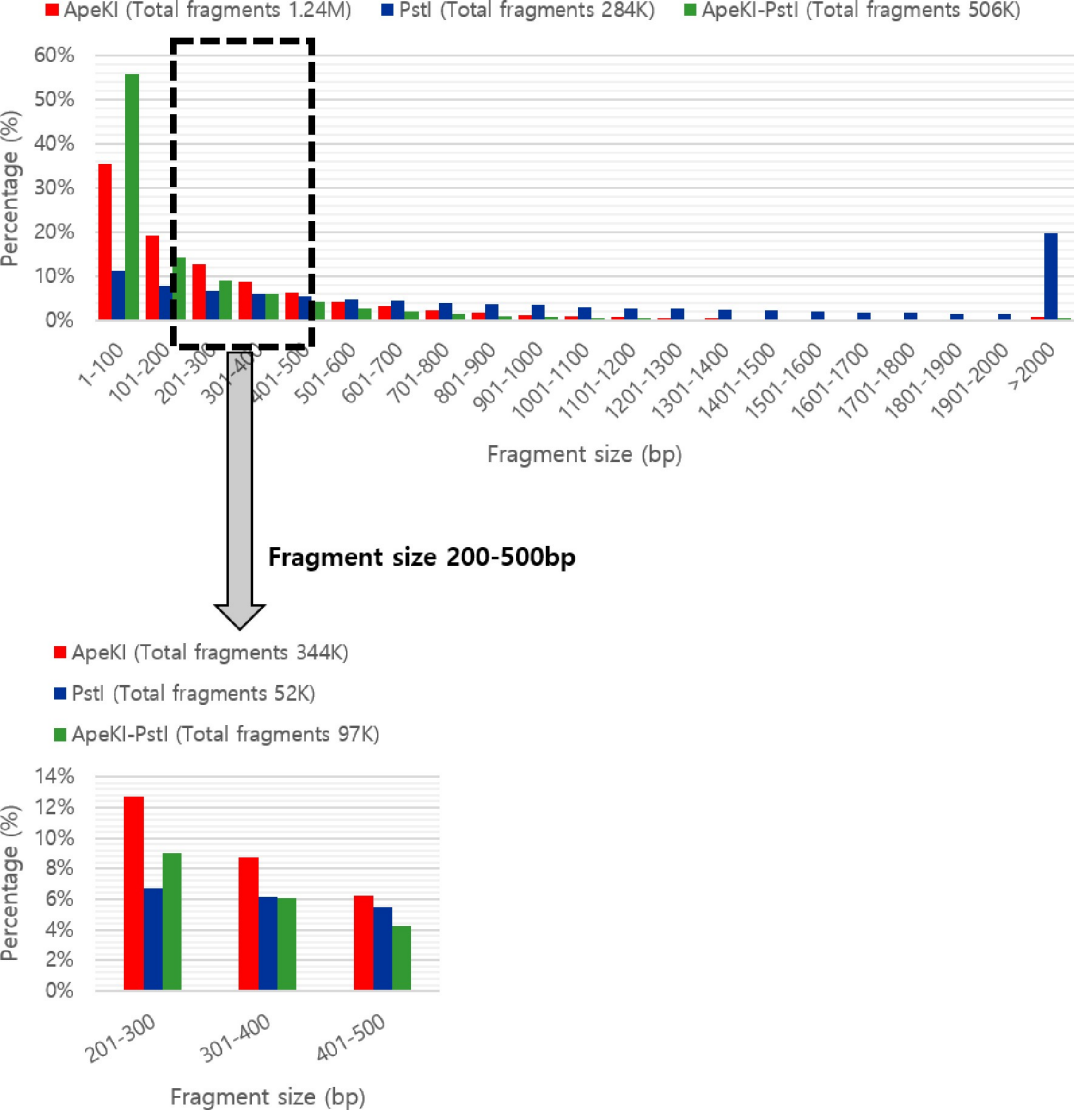
*In silico* analyses of restriction enzyme sites in the *T*. *rubripes* sequence. Fragment size distribution obtained by *in silico* digestion of *Ape*KI, *Pst*I, and *Ape*KI-*Pst*I restriction enzymes showing a higher percentage of *Ape*K1 fragments in a suitable range for genotyping by sequencing.

To obtain restriction fragments of 200–500 bp, we performed in *silico* enzymatic digestion of the *T*. *rubripes* genome using *Ape*KI, *Pst*I, and *Ape*KI-*Pst*I. *Ape*KI was predicted to generate 344 K fragments, *Pst*I 52 K fragments, and *ApeKI-Pst*I 97 K fragments (Fig 1). Therefore, we conducted in *silico* digestion using *Ape*KI (GCWGC) with a protocol modified from that of Elshire et al. [17].

Sequencing on the Illumina Hiseq 2500 platform generated 573,121,290 paired-end unique reads and 57,885,250,290 bp. The number of raw data reads for each sample after de-multiplexing using the barcode sequence was 537,661,624 (93.81%). After de-multiplexing, removing the barcode and adaptor sequences, and quality trimming, the number of reads mapped to the reference genome was 425,121,518, which accounted for >90% of the annotated genes.

### Selection of useful SNPs and phylogenetic analysis

**Fig 2:**
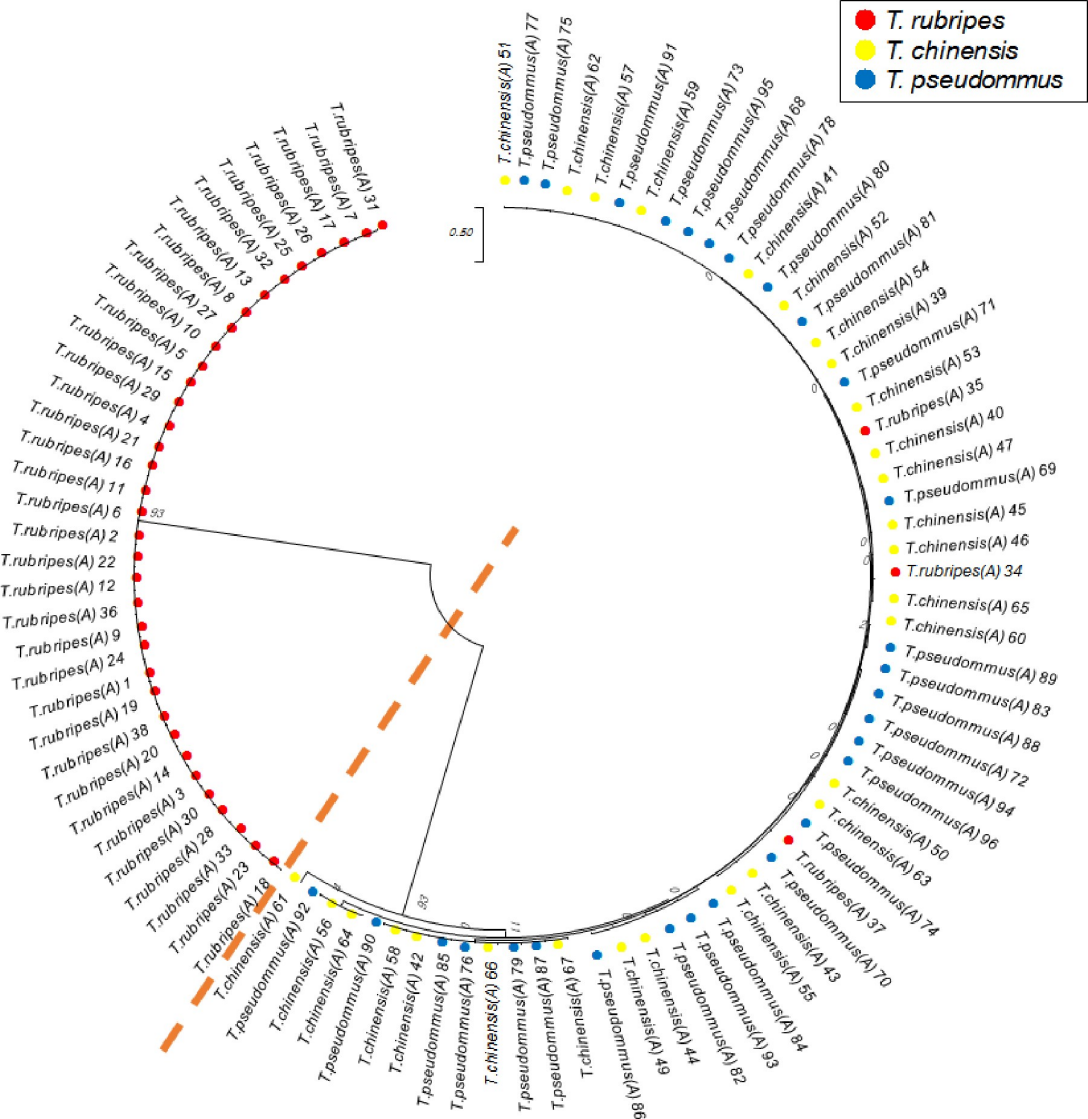
Phylogenetic relationships of sample set A among *T*. *rubripes*, *T*. *chinensis*, and *T*. *pseudommus*. Red circle, *T*. *rubripes*; yellow circle, *T*. *chinensis*; blue, *T*. *pseudommus*; dotted line, separation between clades.

**Fig 3:**
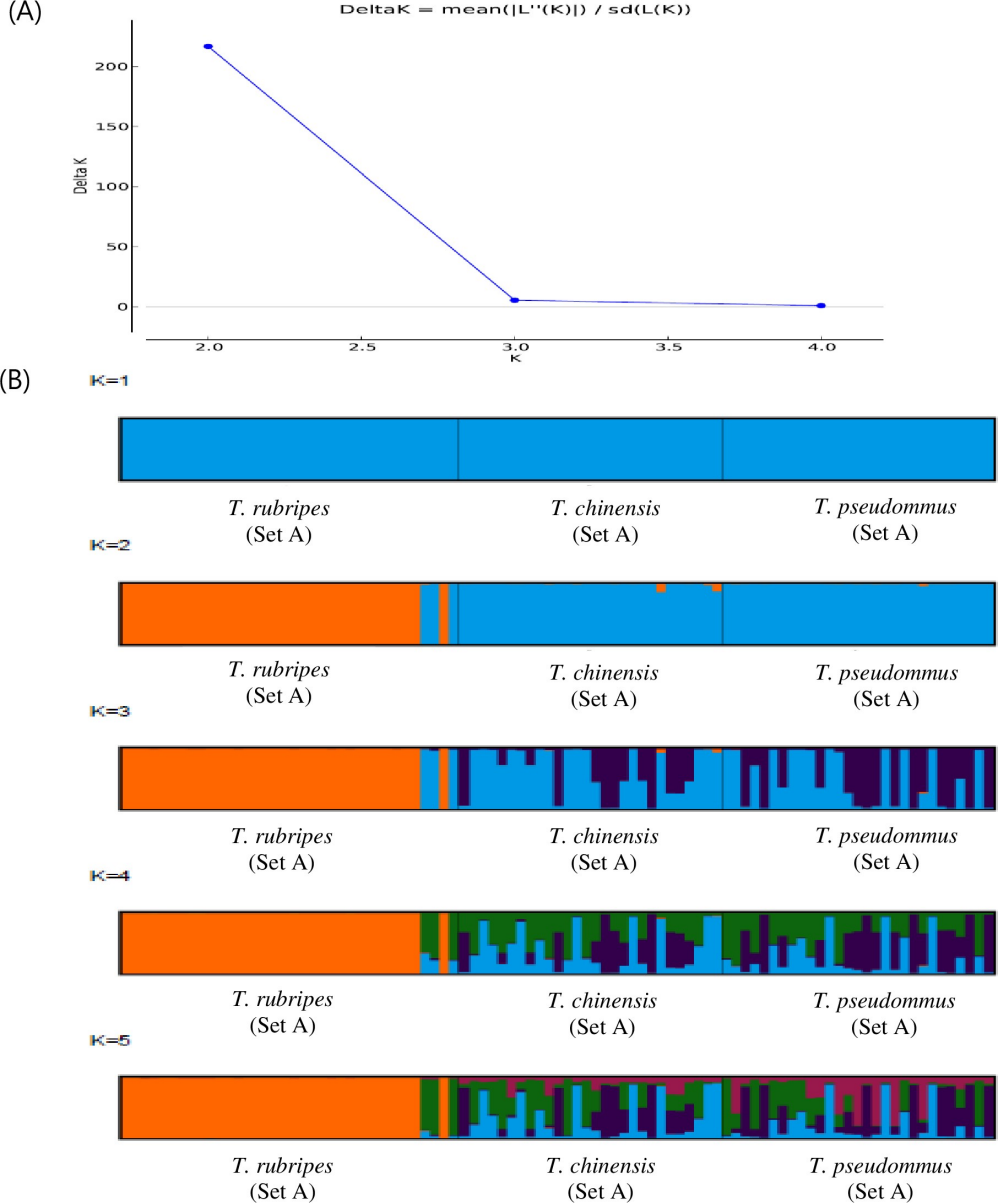
Gene pool classification of the genus *Takifugu* assessed by STRUCTURE. (A) Mean absolute difference of the second order rate of change with respect to K. K (K = 2) indicates the number of clusters that maximized the probability of the model. (B) An STRUCTURE analyses of a polymorphic SNP dataset of individuals of *T*. *rubripes*, *T*. *chinensis*, and *T*. *pseudommus* to evaluate the population structures of these three species.

In all, 638,028 SNP matrix loci were combined using the raw SNPs of each sample including 96 of the genus *Takifugu*. These SNP markers were subjected to a filtration for MAF and missing data and 16,577 loci with MAF> 10% and <30% missing data were obtained. These SNPs were analyzed common SNP loci referring SNP location common within the same species in integrated SNP matrix of the 96 sample, resulted in 210,656 of *T*. *rubripes*, 141,481 of *T*. *chinensis* and 117,348 of *T*. *pseudomonas* and 159,284 comparable common SNP loci were selected. Among these, 18 polymorphic SNP loci which showed the interspecies polymorphism at the same SNP location among *Takifugu* were selected. Eighteen SNPs were used to construct a maximum-likelihood phylogenetic tree (Fig 2) with 1,000 bootstraps and a 70% cut-off value among the *Takigufu* groups. *T*. *chinensis* and *T*. *pseudommus* formed one clade. The *T*. *rubripes* clade was separated into their respective taxa excluded three individuals which included sample of *T*. *rubripes* 34, 35, and 37 from Sinan and Incheon (the circle of Red in Fig 2). To analyze genetic differentiation confirmed the results exhibited by STRUCTURE outputs predicted *K* = 2 as the most likely value of clusters to distinguish *T*. *rubripes* clade (Fig 3A). STRUCTURE analyses based on all individuals showed that *T*. *chinensis* and *T*. *pseudommus* belong to one cluster while *T*. *rubripes* formed a separate cluster(Orange), with the exception of three individuals (Fig 3B). This indicates that *T*. *rubripes* could be differentiated from *T*. *chinensis* and *T*. *pseudommus*. Next, we validated these data by Sanger sequencing using sample set B.

### Experimental validation of SNPs

**Fig 4:**
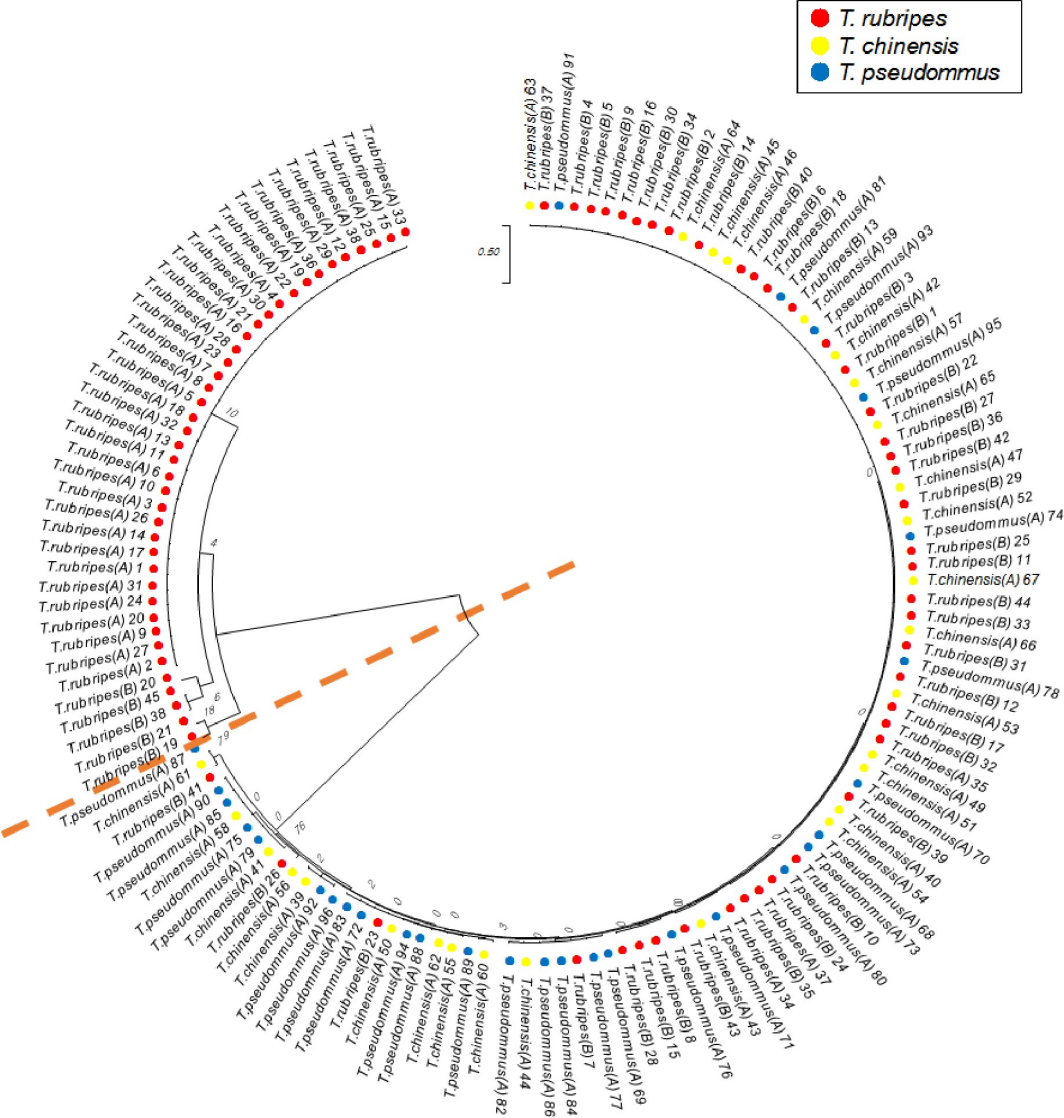
To validate the 18 polymorphic SNPs, a phylogenetic tree was constructed using sample set A and B. Red circle, *T*. *rubripes*; yellow circle, *T*. *chinensis*; blue, *T*. *pseudommus*; dotted line, separation between clades.

**Fig 5:**
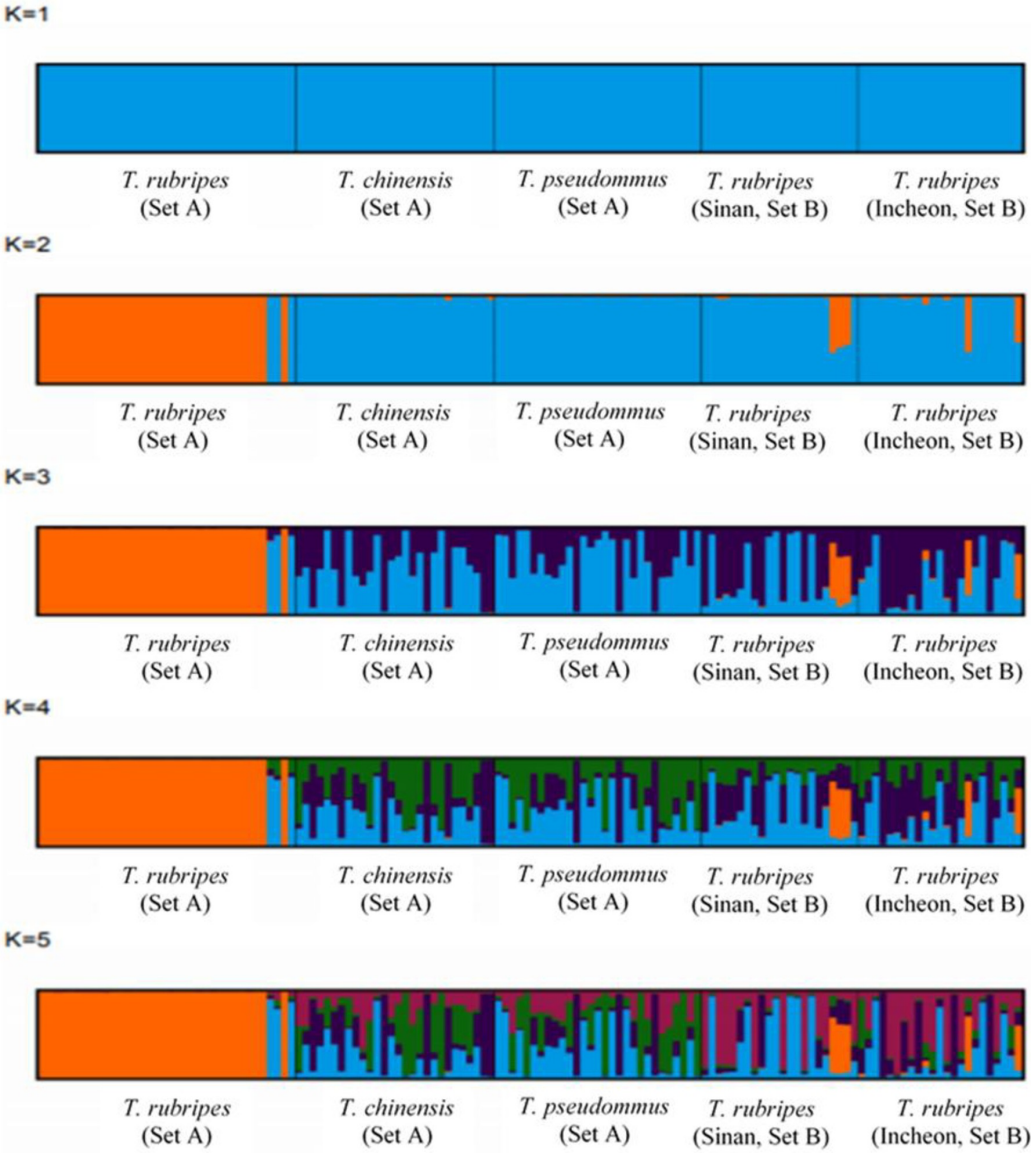
STRUCTURE analyses of sample sets A and B. Bar plots for each value of *K*, *K* = 2 indicate the number of clusters that maximized the probability of the model. Each individual is represented by a vertical bar.

Eighteen SNP loci identified based on the GBS library were amplified for sample set B. Eighteen SNP details and information were shown in Table 3. These loci were validated from 45 individuals by Sanger sequencing. A phylogenetic tree of the 18 SNPs was produced using the reference sequence of set A and the validation sequence of set B. The tree indicated that 89% of all individuals identified as *T*. *rubripes* in set B were grouped with *T*. *chinensis* and *T*. *pseudommus* (Fig 4). This is in contrast with the grouping of only three *T*. *rubripes* individuals in the *T*. *chinensis* and *T*. *pseudommus* clade. STRUCTURE analyses showed that the *T*. *rubripes* cluster of set B was not separated from *T*. *chinensis* and *T*. *pseudommus* (Fig 5).

**Table 3:** Sequence of primers used to amplify the 18 polymorphic SNPs.

No.	Name	Forward (5’→3’)	Reverse (5’→3’)	SNP position
1	Taki-G-01	CACCACAGCACACAGACATC	CACACAGCTACTTGACATCAGG	297
2	Taki-A-02	CCTTCATGCACACGTCTTCC	AGCCCACCAAACTGTGACTA	318
3	Taki-G-03	CGTCACCGTTTCTTCAAGG	CAGGAGTCAGCATACCAAAG	250
4	Taki-T-04	TGGGAGCTGATGAAAGACC	CTGGCCTTCCGAACCTTG	235
5	Taki-C-05	CCAGAAGGTGGTCCAGACAT	GTGTGTGTGTGTGTGTGTGT	259
6	Taki-G-06	TGGTGACTTTACCCTGGGAC	CTAACACCAACACCAAGGGC	244
7	Taki-A-07	CTTCCTCTTCTCCTTCCCGG	CTTCAGCCAGGGTGAGAGAA	352
8	Taki-G-08	TGTGATGAGGGTAAAAACGG	ATCCCCCAGTAGTTGTTCC	291
9	Taki-A-09	ACCACCTATTTAGCCTCCCG	CTAGCACCAATCTGCCAACC	270
10	Taki-C-10	CTCCTAAACGGCGCTTTACC	TGTCGTTTGTCTCCCACAGA	303
11	Taki-C-11	TCAAGCGGAAAACCAACAGG	GCACGCATCATAAGCTCCTC	322
12	Taki-A-12	CAGAGCCTTGATGCCACTTC	CTCCGTGCTGGTATCAGAGT	231
13	Taki-A-13	TTGAGATCGGTTCCCACCAA	GTTGCTTTCAACGCGTTTAA	276
14	Taki-G-14	GGAGCTCCTGGGGGTATTAC	ACTCTCTCTCTGCCCCTCTC	329
15	Taki-A-15	GAGCGTGTCATTACCTGCTG	ATGGCAGGGAAGTCAAAACG	321
16	Taki-C-16	TGTGACATTCCTGGGGTCTC	AAGGTGTGTGACACTTTGGC	266
17	Taki-A-17	GCCAAAATGTATTACCTGCAGC	ATGCTGCACAAACTCCCATG	321
18	Taki-T-18	CTTGGAGGAAGGCAGACAGA	GTGTGTGCGGAAATGAGTGT	300

## Discussion

SNPs are the most frequently used polymorphisms for simultaneous identification and genotyping [32]. Genome-wide SNP discovery may be performed using GBS, restriction-associated DNA sequencing, or reduced representation libraries. GBS offers ultra-high-throughput sequencing, requires a small amount of DNA (100 ng), and enables a high level of multiplexing [33]. Selection of appropriate restriction enzymes is important for GBS because this influences the properties of the libraries and the extent of SNP coverage [34]. The initial protocol involved one enzyme and was subsequently modified to use two [35]. A two-enzyme GBS protocol reduces genome complexity by avoiding sequencing of repetitive regions and generates a more uniform library for sequencing than a one-enzyme GBS protocol [36]. Therefore, we performed genome digestion using both one- and two-enzyme protocols. The restriction enzyme was selected based on a prior report [34]. Using only a single enzyme, *Ape*KI yielded more fragments than the other enzymes (Fig 1). In addition, use of *Ape*KI resulted in identification of more SNPs than the other restriction enzymes, but with an imbalanced density distribution, probably because of its sensitivity to methylation [37]. GBS typically results in a large amount of missing data because of its limited sequencing depth [38]. To increase data quality, Liu et al. [38] proposed modulation of high-quality SNPs with < 20% missing data. However, this approach was at risk of loss of some SNPs, so Lin et al [39] suggested <50% missing data. Therefore, common SNPs were composed of data having less than 50% missing values.

GBS-SNP could simplify the alignment problem in species with a high level of genetic diversity. Indeed, GBS is suitable for population diversity, germplasm characterization, breeding, and trait mapping in diverse organisms [17]. In addition, it can resolve phylogenetic relationships and genetic diversity in tomato, rice, and lentil [40, 41]. In our study, 18 polymorphic SNPs were identified to evaluate the genetic relationships among three *Takifugu* species. Phylogenetic analyses based on all individuals of set A indicated that the *T*. *rubripes* clade, with the exception of three individuals from Sinan, Incheon, was distributed in the *T*. *chinensis* and *T*. *pseudommus* clade (Fig 2). STRUCTURE analyses using the same individuals yielded the same result (Fig 3B). Most *T*. *rubripes* individuals of set A were from aquaculture in Japan; the remaining individuals were caught wild in the ROK and from culture in China, including three individuals in the *T*. *chinensis* and *T*. *pseudommus* clade (Fig 2). Therefore, the three *T*. *rubripes* individuals from Sinan, Incheon, which were grouped in the *T*. *chinensis* and *T*. *pseudommus* clade, have a genetically complex structure or unclear phenotype, such as an interspecies hybrid. Indeed, one interspecies hybrid was 99–100% genetically identical to *T*. *rubripes*, *T*. *chinensis*, and *T*. *pseudommus* and had morphological features of both *T*. *rubripes* and *T*. *chinensis* [2]. In addition, we speculate that most *T*. *rubripes* in set A were from aquaculture in Japan, and so had low genetic diversity. Thus, *T*. *rubripes* was genetically different from *T*. *chinensis* and *T*. *pseudommus*. All of the cultured *T*. *rubripes* individuals were of one haplotype in COI region (Table 2). Cui et al. [14] reported that cultured *T*. *rubripes* had lower genetic diversity (0.7832) than wild *T*. *rubripes* and *T*. *pseudommus* (0.8661 and 0.8272, respectively).

We used sample set B to validate the ability of the polymorphic SNPs to evaluate genetic variation among the three *Takifugu* species. Unlike the results obtained using sample set A (Fig 2), most *T*. *rubripes* in set B grouped with the *T*. *chinensis* and *T*. *pseudommus* clade (Figs 4 and 5). This indicates no genetic variation among *T*. *rubripes*, *T*. *chinensis*, and *T*. *pseudommus*. A variety of DNA-based markers, such as microsatellites, mitochondrial DNA, and randomly amplified polymorphic DNA (RAPD), have been used to evaluate the genetic relationships among *T*. *rubripes*, T. chinensis, and *T*. *pseudommus*. Reza et al. [7] performed sequence analyses of mitochondrial and nuclear genes of *T*. *rubripes* and *T*. *chinensis*, and the results indicated that both are of the same species but have different phenotypes. In addition, Reza et al. [42], using microsatellites and the mitochondrial control region, reported that *T*. *rubripes* and *T*. *chinensis* could be considered the same species. In addition, RAPD, mitochondrial 16S ribosomal RNA gene sequences, and microsatellites have indicated that *T*. *rubripes* and *T*. *pseudommus* are genetically similar [43]. Moreover, Baek et al. [2] reported that the COI sequence of *T*. *rubripes*, *T*. *chinensis*, *T*. *pseudommus*, and an interspecies hybrid were 99–100% similar. Therefore, further studies of the genetic relationships among the three *Takifugu* species are warranted. we could not identify the genetic difference among the three species of *Takifugu* species, whole genome sequence analysis by NGS might be necessary for this purpose. In this study, we used GBS-SNP to assess the genetic relationships among *T*. *rubripes*, *T*. *chinensis*, and *T*. *pseudommus*. Our results suggest that these species exhibit a very low level of genetic variation and could be considered one species, in agreement with previous reports.

All 18 SNPs were selected because they were polymorphic in three *Takifugu* species and expected to differentiate the species. However, the validation process showed that these three *Takifugu* species could not be differentiate as in case of COI comparison. Therefore, there is not species specific marker until now and further study for molecular maker is need or these three species could be regard as subspecies of one species as remarked in the discussion.

## References

[1] Kim IS, Lee WO. Synopsis of the suborder Tetraodontoidei (Pisces; Tetraodontiformes) from Korea. Korea J Ichtyol. 1990; 2: 1–27.

[2] Baek JI, Han KH, Lee SH, Kim JK. Morphological and molecular comparison among three species and one unidentified Takifugu species. Korean J Fish Aquat Sci. 2018; 51: 404–410.

[3] Hwang SM, Oh KS. Comparisons of food component characteristics of wild and cultured edible pufferfishes in Korea. Korean J Fish Aquat Sci. 2013; 46: 725–732.

[4] Cui JZ, Shen XY, Gong QL, Yang GP, Gu QQ. Identification of sex markers by cDNA-AFLP in *Takifugu rubripes*. Aquaculture. 2006; 257: 30–36.

[5] Cheng QT, Wang CX, Tian MC, Li CS, Wang YG, Wang Q. Studies on the Chinese tetraodonoid fishes of the genus Fugu. Acta Zool Sinica. 1975; 21: 359–378.

[6] Nakabo T. Fishes of Japan, with Pictorial Keys to the Species. English edition II. Tokai University Press, Tokyo 2002; 1425–1426.

[7] Reza S, Furukawa S, Mochizuki T, Matsumura H, Watabe S. Genetic comparison between torafugu *Takifugu rubripes* and its closely related species karasu *Takifugu chinensis*. Fisheries science. 2008; 74: 743–754.

[8] Chuhan T, Rajiv K. Molecular markers and their applications in fisheries and aquaculture. Adv Biosci Biotechnol. 2010; 1: 281–291.

[9] Elliott NG, Ward RD. Genetic relationships of eight species of Pacific tunas (Teleostei: Scombridae) inferred from allozyme analysis. Mar Freshw Res. 1995; 46: 1021–1032.

[10] Park YJ, Lee MN, Kim EM, Noh ES, Noh JK, Park JY, et al Development of the duplex-PCR method of identifying *Trachurus japonicus* and *Trachurus novaezelandiae*. J Life Sci. 2018; 28: 1062–1067.

[11] Lakra WS, Goswami M, Mohindra V, Lal KK, Punia P. Molecular identification of five Indian sciaenids (pisces: perciformes, sciaenidae) using RAPD markers. Hydrobiologia, 2007; 583: 359–363.

[12] Di Finizio A, Guerriero G, Russo GL, Ciarcia G. Identification of gadoid species (Pisces, Gadidae) by sequencing and PCR–RFLP analysis of mitochondrial 12S and 16S rRNA gene fragments. Eur Food Res Technol. 2007; 225: 337.

[13] Tautz D. Hypervariability of simple sequence as a general source for polymorphic DNA marker. Nucleic Acids Res. 1989; 17: 6463–6471. 10.1093/nar/17.16.6463 2780284 PMC318341

[14] Cui JZ, Shen XY, Yang GP, Gong QL, Gu QQ. Characterization of microsatellite DNAs in *Takifugu rubripes* genome and their utilization in the genetic diversity analysis of *T*. *rubripes* and *T*. *pseudommus*. Aquaculture. 2005; 250: 129–137.

[15] Vignal A, Milan D, SanCristobal M, Eggen A. A review on SNP and other types of molecular markers and their use in animal genetics. Genet Sel Evol. 2002; 34: 275–306. 10.1186/1297-9686-34-3-275 12081799 PMC2705447

[16] Zhu YL, Song QJ, Hyten DL, Van Tassell CP, Matukumalli LK, Grimm DR, et al Single-nucleotide polymorphism in soybean. Genetics 2003; 163: 1123–1134. 12663549 10.1093/genetics/163.3.1123 PMC1462490

[17] Elshire RJ, Glaubitz JC, Sun Q, Poland JA, Kawamoto K, Buckler ES, et al A robust, simple genotyping-by-sequencing (GBS) approach for high diversity species. PLoS One. 2011; 6: e19379 10.1371/journal.pone.0019379 21573248 PMC3087801

[18] Davey JW, Hohenlohe PA, Etter PD, Boone JQ, Catchen JM, Blaxter ML. Genome-wide genetic marker discovery and genotyping using next-generation sequencing. Nat Rev Genet. 2011; 12: 499–510. 10.1038/nrg3012 21681211

[19] Glazer AM, Killingbeck EE, Mitros T, Rokhsar DS, Miller CT. Genome assembly improvement and mapping convergently evolved skeletal traits in sticklebacks with genotyping-by-sequencing. G3. 2015; g3–115. 10.1534/g3.115.017905 PMC4502380 26044731

[20] Larson WA, Seeb JE, Pascal CE, Templin WD, Seeb LW. Single-nucleotide polymorphisms (SNPs) identified through genotyping-by-sequencing improve genetic stock identification of Chinook salmon (Oncorhynchus tshawytscha) from western Alaska. Can J Fish Aquat Sci. 2014; 71: 698–708.

[21] Kim JE, Oh SK, Lee JH, Lee BM Jo SH. Genome-Wide SNP calling using next generation sequencing data in tomato. Mol Cells. 2014; 37: 36–42. 10.14348/molcells.2014.2241 24552708 PMC3907006

[22] Martin M. Cutadapt removes adapter sequences from high-throughput sequencing reads. EMBnet J. 2011; 17: 10–12.

[23] Cox MP, Peterson DA, Biggs PJ. SolexaQA: At-a-glance quality assessment of Illumina second-generation sequencing data. BMC Bioinformatics. 2010; 11: 485 10.1186/1471-2105-11-485 20875133 PMC2956736

[24] Li H, Durbin R. Fast and accurate short read alignment with Burrows-Wheeler transform. Bioinformatics. 2009; 25: 1754–1760. 10.1093/bioinformatics/btp324 19451168 PMC2705234

[25] Li H, Handsaker B, Wysoker A, Fennell T, Ruan J, Homer N, et al The sequence alignment/map format and SAMtools. Bioinformatics. 2009; 25: 2078–2079. 10.1093/bioinformatics/btp352 19505943 PMC2723002

[26] Eun MH, Han JH, Yoon JB, Lee J. QTL mapping of resistance to the Cucumber mosaic virus P1 strain in pepper using a genotyping-by-sequencing analysis. Hortic Environ Biotechnol. 2016; 57: 589–597.

[27] Zhu B, Zhang JJ, Niu H, Guan L, Guo P, Xu L Y, et al (2017). Effects of marker density and minor allele frequency on genomic prediction for growth traits in Chinese Simmental beef cattle. J Integr Agric. 2017; 16: 911–920.

[28] Untergasser A, Cutcutache I, Koressaar T, Ye J, Faircloth BC, Remm M, et al Primer3—new capabilities and interfaces. Nucleic acids research. 2012; 40: e115–e115. 10.1093/nar/gks596 22730293 PMC3424584

[29] Librado P, Rozas J. DnaSP v5: a software for comprehensive analysis of DNA polymorphism data. Bioinformatics. 2009; 25: 1451–1452. 10.1093/bioinformatics/btp187 19346325

[30] Tamura K, Stecher G, Peterson D, Filipski A, Kumar S. MEGA6: molecular evolutionary genetics analysis version 6.0. Mol Biol Evol. 2013; 30: 2725–2729. 10.1093/molbev/mst197 24132122 PMC3840312

[31] Pritchard JK, Stephens M, Donnelly P. Inference of population structure using multilocus genotype data. Genetics. 2000; 155: 945–959. 10835412 10.1093/genetics/155.2.945 PMC1461096

[32] Rafalski A. Applications of single nucleotide polymorphism in crop genetics. Curr Opin Plant Biol. 2002; 5: 94–100. 10.1016/s1369-5266(02)00240-6 11856602

[33] Zargar SM, Raatz B, Sonah H, Bhat JA, Dar ZA, Agrawal GK, et al Recent advances in molecular marker techniques: insight into QTL mapping, GWAS and genomic selection in plants. J Crop Sci Biotech. 2015; 18: 293–308.

[34] Hamblin MT, Rabbi IY. The effects of restriction-enzyme choice on properties of genotyping-by-sequencing libraries: a study in cassava (Manihot esculenta). Crop Science. 2014; 54: 2603–2608.

[35] Sonah H, Bastien M, Iquira E, Tardivel A, Legare G, Boyle B, et al An improved genotyping by sequencing (GBS) approach offering increased versatility and efficiency of SNP discovery and genotyping. PLoS One. 2013; 8: e54603 10.1371/journal.pone.0054603 23372741 PMC3553054

[36] He J, Zhao X, Laroche A, Lu ZX, Liu H, Li Z. Genotyping-by-sequencing (GBS), an ultimate marker-assisted selection (MAS) tool to accelerate plant breeding. Front Plant Sci. 2014; 5: 484 10.3389/fpls.2014.00484 25324846 PMC4179701

[37] Carrasco B, González M, Gebauer M, García-González R, Maldonado J, Silva H. Construction of a highly saturated linkage map in Japanese plum (Prunus salicina L.) using GBS for SNP marker calling. PloS ONE. 2018; 13: e0208032 10.1371/journal.pone.0208032 30507961 PMC6277071

[38] Liu H, Bayer M, Druka A, Russell JR, Hackett CA, Poland J, et al An evaluation of genotyping by sequencing (GBS) to map the Breviaristatum-e (ari-e) locus in cultivated barley. BMC genomics. 2014; 15: 104–114. 10.1186/1471-2164-15-104 24498911 PMC3922333

[39] Lin M, Cai S, Wang S, Liu S, Zhang G, Bai G. Genotyping-by-sequencing (GBS) identified SNP tightly linked to QTL for pre-harvest sprouting resistance. Theor Appl Genet. 2015; 128: 1385–1395. 10.1007/s00122-015-2513-1 25851002

[40] Labate JA, Robertson LD, Strickler SR, Mueller LA. Genetic structure of the four wild tomato species in the *Solanum peruvianum* s.l. species complex. Genome. 2014; 57: 169–180. 10.1139/gen-2014-0003 24884691

[41] Escudero M, Eaton DA, Hahn M, Hipp AL. Genotyping-by-sequencing as a tool to infer phylogeny and ancestral hybridization: A case study in Carex (Cyperaceae). Mol Phylogenet Evol. 2014; 79: 359–367. 10.1016/j.ympev.2014.06.026 25010772

[42] Reza MS, Kinoshita S, Furukawa S, Mochizuki T, Watabe S. Microsatellite and mitochondrial DNA analyses reveal no genetic difference between two pufferfish species torafugu *Takifugu rubripes* and karasu *T*. *chinensis*. Fish Sci. 2011; 77: 59–67.

[43] Song L, Liu B, Xiang J, Qian PY. Molecular phylogeny and species identification of pufferfish of the genus Takifugu (Tetraodontiformes, Tetraodontidae). Mar biotechnol. 2001; 3: 398–406. 10.1007/s10126-001-0006-5 14961355

